# Direct electrochemical analyses of human cytochromes *b*_5 _with a mutated heme pocket showed a good correlation between their midpoint and half wave potentials

**DOI:** 10.1186/1423-0127-17-90

**Published:** 2010-12-04

**Authors:** Tomomi Aono, Yoichi Sakamoto, Masahiro Miura, Fusako Takeuchi, Hiroshi Hori, Motonari Tsubaki

**Affiliations:** 1 Department of Chemistry, Graduate School of Science, Kobe University, 1-1 Rokkodai-cho, Nada-ku, Kobe, Hyogo 657-8501, Japan; 2 Department of Pharmacy, College of Pharmaceutical Sciences, Ritsumeikan University, Kusatsu, Shiga 525-8577, Japan; 3 Center for Quantum Science and Technology under Extreme Conditions, Osaka University, 1-3 Machikaneyama-cho, Toyonaka, Osaka 560-8531, Japan

## Abstract

**Background:**

Cytochrome *b*_5 _performs central roles in various biological electron transfer reactions, where difference in the redox potential of two reactant proteins provides the driving force. Redox potentials of cytochromes *b*_5 _span a very wide range of ~400 mV, in which surface charge and hydrophobicity around the heme moiety are proposed to have crucial roles based on previous site-directed mutagenesis analyses.

**Methods:**

Effects of mutations at conserved hydrophobic amino acid residues consisting of the heme pocket of cytochrome *b*_5 _were analyzed by EPR and electrochemical methods. Cyclic voltammetry of the heme-binding domain of human cytochrome *b*_5 _(HLMW*b*_5_) and its site-directed mutants was conducted using a gold electrode pre-treated with β-mercarptopropionic acid by inclusion of positively-charged poly-L-lysine. On the other hand, static midpoint potentials were measured under a similar condition.

**Results:**

Titration of HLMW*b*_5 _with poly-L-lysine indicated that half-wave potential up-shifted to -19.5 mV when the concentration reached to form a complex. On the other hand, midpoint potentials of -3.2 and +16.5 mV were obtained for HLMW*b*_5 _in the absence and presence of poly-L-lysine, respectively, by a spectroscopic electrochemical titration, suggesting that positive charges introduced by binding of poly-L-lysine around an exposed heme propionate resulted in a positive shift of the potential. Analyses on the five site-specific mutants showed a good correlation between the half-wave and the midpoint potentials, in which the former were 16~32 mV more negative than the latter, suggesting that both binding of poly-L-lysine and hydrophobicity around the heme moiety regulate the overall redox potentials.

**Conclusions:**

Present study showed that simultaneous measurements of the midpoint and the half-wave potentials could be a good evaluating methodology for the analyses of static and dynamic redox properties of various hemoproteins including cytochrome *b*_5_. The potentials might be modulated by a gross conformational change in the tertiary structure, by a slight change in the local structure, or by a change in the hydrophobicity around the heme moiety as found for the interaction with poly-L-lysine. Therefore, the system consisting of cytochrome *b*_5 _and its partner proteins or peptides might be a good paradigm for studying the biological electron transfer reactions.

## Background

Cytochromes *b *can be defined as electron transfer proteins having heme *b *group(s), noncovalently bound to the protein. *b*-Type cytochromes possess a wide range of properties and functions in a large number of different redox processes. Among them, cytochromes *b*_5 _are ubiquitously found in animals, plants, fungi and some bacteria. The microsomal and mitochondrial (outer membrane; OM) variants are known and are present in a membrane-bound form. On the other hand, bacterial and those from erythrocytes and some animal tissues are water-soluble (such as for the reduction of methemoglobin in erythrocytes and for the biosynthesis of *N*-glycolylneuraminic acid [1]). A membrane-bound (microsomal) form of cytochrome *b*_5 _is required for numerous biosynthetic and biotransformation reactions, which include cytochrome P450-dependent reactions [2], desaturation of fatty acids [3], plasmalogen biosynthesis [4], and cholesterol biosynthesis [5,6]. The role of cytochrome *b*_5 _in microsomal P-450-dependent monooxygenase reactions has been studied most extensively [2]. In addition, a number of fusion enzymes exist in nature containing cytochrome *b*_5 _as a domain component. These include mitochondrial flavocytochrome *b*_2 _(L-lactate dehydrogenase) [7], sulfite oxidase [8], the ∆^5^- and ∆^6^-fatty acid desaturases [9], and yeast inositolphosphorylceramide oxidase [10]. Plant and fungal nitrate reductases are also cytochrome *b*_5_-containing fusion enzymes [11].

For human cytochrome *b*_5_, only a few naturally occurring mutations recognized as a genetic disorder have been reported. One such example was found by Kurian *et al*. [12]. They reported that naturally occurring human cytochrome *b*_5 _T60A mutant [12] displayed an impaired hydroxylamine reduction capacity. They observed further that the expressed protein in rabbit reticulocyte lysate system showed an enhanced susceptibility to the proteolytic degradation. Expression level in transfected HeLa cells was also significantly lowered. Another genetically confirmed example was previously reported. In this case, Steggles *et al*. identified a homozygous splice site mutation in the CYB5A gene, resulting in premature truncation of the protein, leading to a very high methemoglobin concentration in red blood cells of the patient, being consistent with methemoglobinemia type IV [13]. The patient exhibited female genitalia at birth, but, was determined as a male pseudohermaphrodite, probably due to the low levels of androgen synthesis by the lack of cytochrome *b*_5 _activity, which has been shown to participate in 17α-hydroxylation in adrenal steroidogenesis [14].

Whereas more than 300 patients had been reported with hereditary methemoglobinemia types I or II, only a few cases of type IV had been reported. Thus, one may attribute that the rarity of naturally occurring cytochrome *b*_5 _mutation may be due to lethality of most type IV mutations. However, in a very recent study by employing transgenic mice, Finn *et al*. found that cytochrome *b*_5 _completely null mice were viable, fertile and produced grossly normal pups at expected Mendelian ratios [15]. Further, the cytochrome *b*_5 _null mice exhibited a number of intriguing phenotypes, including altered drug metabolism, methemoglobinemia, disrupted steroid hormone biosynthesis. In addition, the cytochrome *b*_5 _null mice displayed skin defects and retardation of neonatal development. These observations suggested that cytochrome *b*_5 _might play a role controlling saturated/unsaturated homeostasis of fatty acids in higher animals including human.

**Figure 1:**
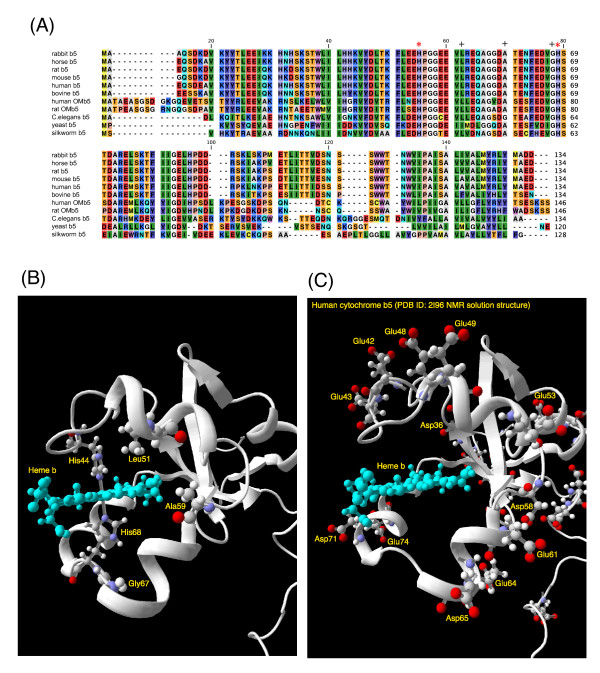
**Alignment of amino acid sequences of cytochrome *b*_5 _from various species (A), a close-up view of tertiary structure of human cytochrome *b*_5 _around the heme-pocket with three conserved hydrophobic residues (Leu51, Ala59, and Gly67) and two heme axial ligands (His44 and His68) indicated (B), a close-up view around the heme pocket with acidic amino acid residues (C)**. (**A**) Amino acid sequences of cytochromes *b*_5 _from various species are aligned. Two heme axial ligands (His44 and His68) are indicated by an asterisk (*). On the other hand, corresponding positions to three target residues (Leu51, Ala59, and Gly67) in the present study are indicated by a cross (+). Amino acid sequences were obtained from [GenBank; NP_001164735 for rabbit *b*_5_, P00170 for horse *b*_5_, AAB67610 for rat *b*_5_, P56395 for mouse *b*_5_, AAA35729 for human *b*_5_, NP_776458 for bovine *b*_5_, BAA23735 for human OM*b*_5_, AAH72535 for rat OM*b*_5_; CAB01732 for *C.elegans b*_5_, P40312 for yeast *b*_5_, NP_001106739 for silkworm *b*_5_]. (**B**) Human cytochrome *b*_5 _NMR solution structure [PDB code: 2I96 model 1] is shown in a ribbon model with a bound heme *b *prosthetic group. In addition, three conserved residues (Leu51, Ala59, and Gly67) and two heme axial ligands (His44 and His68) are indicated. (**C**) Acidic amino acid residues located on the surface of the heme-binding domain (corresponding to LMW*b*_5_) are indicated.

The membrane-bound form of cytochrome *b*_5 _is associated with the endoplasmic reticulum. It has a molecular weight of 16,700 Da and contains about 134 amino acids in animals (Figure 1A). It is composed of three domains: a hydrophilic heme-containing catalytic domain of about 99 amino acids; a membrane-binding hydrophobic domain containing about 30 amino acids at the carboxy terminus of the molecule; and a membrane-targeting region represented by the 10-amino-acid sequence located at the carboxy-terminus of the membrane-binding domain. Three-dimensional structures of a number of cytochrome *b*_5 _are known [16], but only for the heme-containing hydrophilic catalytic domain [17]. Two His residues (His44 and His68) provide the fifth and sixth heme ligands (Figure 1A, B), and two propionate groups of the heme *b *lies at the opening of the heme-binding pocket, which is formed by highly conserved hydrophobic amino acid residues (Figure 1A). The roles of each amino acid were investigated by detailed site-directed mutagenesis in the past with employing various structural, spectroscopic and electrochemical techniques, including X-ray crystallography [18-20], NMR [21-23], UV-visible absorption spectroscopy, and redox potential measurements [24].

Redox potentials of various forms of cytochrome *b*_5 _span a range of ~400 mV. It is well documented that several factors could regulate and induce changes in the reduction potential of cytochrome *b*_5 _spanning almost entire range observed. The electrostatic contribution by surface charges might play important roles in adjusting the selectivity of the protein-protein interaction. On the other hand, difference in the redox potential of two reactant proteins provides the driving force for the electron transfer reactions. Thus, the clarification of the regulatory mechanism of the redox potentials might be essential for the understanding of the biological electron transfer reactions.

Biological redox potential measurements were usually conducted either by an equilibrating electrochemical method or by employing a dynamic cyclic voltammetry. Common features to all the past voltammetric experiments involving cytochrome *b*_5 _and electrodes pre-treated with various thiol-containing aliphatic acid or related groups are the large difference between the half-wave potential (E_1/2_) and the midpoint potential determined by the equilibrating method [25]. In the case of rat OM cytochrome *b*_5_, its midpoint potential determined by the equilibrating method showed as low as -102 mV; whereas the half-wave potential was found as +8 mV [25]. Similar large positive shifts were reported for bovine liver microsomal cytochrome *b*_5 _(~+31 mV) [26] and chicken liver microsomal cytochrome *b*_5 _(~+40 mV) [27].

The large positive shift (+110 mV) observed for rat OM cytochrome *b*_5 _were attributed to the binding of multivalent cations, such as, poly-L-lysine, which were used for shielding the negatively charged protein surface and negatively-charged electrode surface to facilitate the electron transfer [25]. The difference in the potentials was ascribed, initially, for the binding of multivalent cations to the specific charged residues on the surface of cytochrome *b*_5_, such as Glu and Asp (Figure 1C) [25], leading to the modulation of the heme redox potential differently from that measured by the equilibrating method. Later, however, a carboxylate of an exposed heme propionate group and conserved acidic residues (Glu44, Glu48, Glu56, and Asp60) (Figure 1C) (corresponding to Glu49, Glu53, Glu61, and Asp65, respectively, of human cytochrome *b*_5_) were proposed to be responsible for the specific binding of multivalent cations [28]. The formation of such a complex will result in a neutralization of the charge on the heme propionate and lowering of the dielectric of the exposed heme microenvironment by excluding water from the complex interface. These two factors act synergistically to destabilize the positive charge of the ferric heme with respect to the neutral ferrous heme, leading to a positive shift of the redox potential upon binding of poly-L-lysine [28,29]. This postulation was partly verified by the esterification of the heme propionate groups, leading to the half-wave potential to be independent of the concentration of multivalent cations [28,29].

In the present study, we focused on three conserved hydrophobic amino acid residues (Leu51, Ala59, and Gly67) consisting of the heme-binding pocket (Figure 1A, B). These residues were not investigated previously despite of their higher conservation among the various members of cytochrome *b*_5 _protein family (Figure 1A). Gly67 is located besides the heme axial His residue (His68) and is near the entrance of the heme-pocket crevice (Figure 1B). Leu51 and Ala59, on the other hand, are located in the bottom of the heme pocket (Figure 1B). The former is on the side of His44 residue, the other heme axial ligand. The latter is on the side of His68 residue. These two residues might be essential for the stabilization of the heme prosthetic group in the hydrophobic heme pocket. Therefore, we selected replacing amino acid residues not too hazardous for the maintenance of the heme cavity. Accordingly, we chose Thr, Ile, Ala, Ser residues for the replacement of Leu51, Ala59, and Gly67 residues. We produced and purified site-directed mutants for these three sites, having particular interests in the changes of local structure and hydrophobicity of the heme pocket, which may affect the redox properties of cytochrome *b*_5_. We measured spectroscopic and electrochemical properties (*i.e*., redox potentials were analyzed by an equilibrating method and a cyclic voltammetry technique) of these mutants to clarify the structural and electrochemical importance of the conserved residues.

## Methods

### Construction of the expression plasmid for wild-type and site-directed mutants of HLMW*b*_5_

The gene coding for a soluble domain (amino acid residues from Met1 to Leu99; LMW*b*_5_) of human cytochrome *b*_5 _in pIN3/*b*_5_/2E1/OR plasmid [30,31] was subcloned into pCW_ori _vector as previously described [32]. Then, the *BamH *I-*Hind *III fragment of the pC/LMW*b*_5 _plasmid encoding entire LMW*b*_5 _(amino acid residues from Met1 to Leu99) was inserted into the *BamH *I-*Hind *III site of pBluescript II KS(+) to form a plasmid pBS/LMW*b*_5 _for easier handling upon the site-directed mutagenesis. The nucleotide sequence of the pBS/LMW*b*_5 _plasmid was confirmed with a DNA sequencer (PRISM 3100 Genetic Analyzer, ABI).

The site-directed mutagenesis was conducted using QuikChange Site-Directed Mutagenesis Kit (Stratagene, La Jolla, CA, USA) according to the manufacturer's manual. Following mutagenic primers were used (substituted codons are underlined): for L51I, L51I-R (5'-CCAGCTTGTTCCCTGATAACTTCTTCCCCACC-3') and L51I-F (5'-GGTGGGGAAGAAGTTATCAGGGAACAAGCTGG-3'); for L51T, L51T-R (5'-CCAGCTTGTTCCCTTGTAACTTCTTCCCCACC-3') and L51T-F (5'-GGTGGGGAAGAAGTTACAAGGGAACAAGCTGG-3'); for A59V, A59V-R (5'-CCTCAAAGTTCTCAGTAACGTCACCTCCAGCTTG-3') and A59V-F (5'-CAAGCTGGAGGTGACGTTACTGAGAACTTTGAGG-3'); for A59 S, A59S-R (5'-CAAGCTGGAGGTGACTCTACTGAGAACTTTGAGG-3') and A59S-F (5'-CAAGCTGGAGGTGACTCTACTGAGAACTTTGAGG-3'); for G67A, G67A-R‪(5'-GGCATCTGTAGAGTGCGCGACATCCTCAAAGTTC-3') and G67A-F‪‪ (5'-GAACTTTGAGGATGTCGCGCACTCTACAGATGCC-3'); and for G67 S, G67S-R (5'-GGCATCTGTAGAGTGCGAGACATCCTCAAAGTTC-3') and G67S-F (5'-GAACTTTGAGGATGTCTCGCACTCTACAGATGCC-3'). After the site-directed mutagenesis, transformation, and plasmid preparation, each mutated plasmid (pBS/L51I, pBS/L51T, pBS/A59V, pBS/A59 S, pBS/G67A, pBS/G67S) was treated with *Nde *I and *Hind *III. The each *Nde *I-*Hind *III fragment of pBS/LMW*b*_5 _plasmid and the mutated plasmids was inserted into the *Nde *I-*Hind *III site of pET-28b(+) vector (Novagen, Merck, Darmstadt, Germany) to construct pET/HLMW*b*_5_, pET/L51I, pET/L51T, pET/A59V, pET/A59 S, pET/G67A, and pET/G67 S, respectively, to achieve an efficient expression and an easier purification of a recombinant protein. The pET-28b(+) vector contains a 6x-His-tag moiety at the upstream of the *Nde *I-*Hind *III site and, therefore, gives an additional extension with a sequence of MGSSHHHHHHSSGLVPRGSH at the NH_2_-terminus of the LMW*b*_5 _protein (designated as HLMW*b*_5_, hereafter). Mutations were confirmed with an ABI PRISM 3100 Genetic Analyzer (Applied Biosystems Japan Ltd.) for both types of plasmids prepared from pBS and pET vectors. *Escherichia coli *strain BL21(DE3)pLysS was transformed with pET/HLMW*b*_5 _(or with one of the mutated pET plasmids) and was cultivated in low-salt Luria-Bertani (LB) medium containing 30 μg/ml of kanamycin and 34 μg/ml chloramphenicol at 37°C for pre-culture.

After the pre-culture, HLMW*b*_5 _protein (or each mutant protein) was produced by growing the transformed cells at 37°C in TB medium (12.0 g/L of tryptone, 24.0 g/L yeast extract, 4 ml/L glycerol, 23.1 g/L KH_2_PO_4_, and 125.4 g/L K_2_HPO_4_) in the presence of 30 μg/ml of kanamycin and 34 μg/ml of chloramphenicol. Induction of the protein expression was achieved by addition of 200 μM (final) IPTG when the cells had grown to an O.D. of 0.6 at 600 nm. Then, the incubation temperature was lowered to 26°C. Cells were harvested 48 h after the addition of IPTG and were frozen in liquid nitrogen and stored at -80 °C until use. The thawed cells were mixed with a lysis buffer (20 mM Tris-HCl buffer (pH 8.0) containing 0.5 mM EDTA) and disrupted by the treatment with lysozyme (final, 1 mg/mL) and DNase (final, 50 μg/mL) in the presence of 1 mM of phenylmethylsulfonyl fluoride followed by sonication on ice with a model 250 sonifier (Branson Ultrasonic). The disrupted cells were centrifuged at 26,000 g for 20 min at 4 °C. The supernatant was saved as a crude extract.

Purification of HLMW*b*_5 _was conducted as follows. The crude extract was loaded onto a column of DEAE-Sepharose CL-6B previously equilibrated with 20 mM Tris-HCl (pH 8.0) buffer containing 0.5 mM EDTA. The HLMW*b*_5 _was adsorbed in the column as a reddish band. The column was washed with the same buffer containing 50 mM NaSCN. The adsorbed LMW*b*_5 _was eluted by a linear gradient of NaSCN concentration from 50 to 300 mM in the same buffer. Main fractions were collected based on the SDS-PAGE analysis (12% gel) and absorbance at 414 nm and were concentrated to about 5 mL using an Amicon concentrator and a Millipore membrane (MWCO = 10,000). The concentrated HLMW*b*_5 _was, then, subjected onto an affinity column chromatography with Ni-NTA agarose gel (QIAGEN) previously equilibrated with 50 mM sodium phosphate buffer (pH 8.0) containing 10 mM imidazole and 300 mM NaCl. The column was washed with 50 mM sodium-phosphate buffer (pH 8.0) containing 20 mM imidazole and 300 mM NaCl. Finally, adsorbed HLMW*b*_5 _protein was eluted with 50 mM sodium-phosphate buffer (pH 8.0) containing 250 mM imidazole and 300 mM NaCl and the eluate was collected. Fractions that showed a single protein band on SDS-PAGE were pooled and concentrated, gel-filtrated against 50 mM sodium phosphate buffer (pH 7.0) with PD-10 mini-column (Amersham Bioscience). The full-length form of human cytochrome *b*_5 _was purified according to the procedure as described previously [33]. Concentrations of purified recombinant proteins were determined spectrophotometrically from the absorbance at 423 nm in the dithionite-reduced form using the extinction coefficient of 163 mM^-1^cm^-1 ^[34]. The protein concentration was determined with a modified Lowry method as previously described [35], in which bovine serum albumin was used as a standard.

### EPR spectroscopy

Oxidized HLMW*b*_5 _samples (or mutants in the oxidized form) in 50 mM potassium-phosphate buffer (pH 7.0) were concentrated to about 200 ~500 μM with a 50-mL Amicon concentrator fitted with a membrane filter (Millipore PTTK04110; pore size MWCO = 10,000). For HLMW*b*_5 _and G67A mutant, concentrated poly-L-lysine solution (5 mM; Sigma-Aldrich Japan K.K.; mol. wt. = 1,000~4,000; corresponding to 8~30 lysine residues) was added to make its final concentration as 400 μM. The samples were introduced into EPR tubes and frozen in liquid nitrogen (77 K). EPR measurements were carried out at X-band (9.23 GHz) microwave frequency using a Varian E-109 EPR spectrometer with 100-kHz field modulation. An Oxford flow cryostat (ESR-900) was used for the measurements at 15K. The microwave frequency was calibrated with a microwave frequency counter (Takeda Riken Co., Ltd., Model TR5212). The strength of the magnetic field was determined with an NMR field meter (ECHO Electronics Co., Ltd., Model EFM 2000AX). The accuracy of the g-values was approximately +0.01.

### Cyclic voltammetry

All electrochemical measurements were done as previously described [25,32] using a water-jacketed conical cell that allowed measurements to be made at controlled temperatures using volumes as small as 150 μL. An ALS electrochemical analyzer (model 611A) was used for all measurements. All sample solutions (100 μM, heme basis, in 50 mM sodium phosphate buffer pH 7.0) were purged with Ar gas before use and blanketed with Ar during the electrochemical determinations. For the measurements of the full-length form (1-134 aa) of human cytochrome *b*_5_, 50 mM sodium-phosphate buffer (pH 7.0) containing 0.5% (v/v) Triton X-100 was used as the buffer. The Au electrode was derivatized with 100 mM of 3-mercaptopropionate, as previously described [25,32]. Poly-L-lysine was added to a final concentration of 50~300 μM just before the measurements. Concentration of poly-L-lysine solution was calculated assuming the formal mol. wt. = 4,000. Therefore, actual concentration of poly-L-lysine in the sample solution might be higher than the indicated values. The average of the cathodic and anodic peak potentials was taken as the formal potential. All potentials were measured at 25°C versus an Ag/AgCl electrode with an internal filling solution of 3 M KCl saturated with AgCl and are then converted versus the standard hydrogen potential (SHE).

### Spectroscopic redox titrations

Spectroscopic redox titrations were performed essentially as described by Dutton [36] and Takeuchi [37], using a Shimadzu UV-2400PC spectrometer equipped with a thermostatted cell holder connected to a low temperature thermobath (NCB-1200, Tokyo Rikakikai Co, Ltd, Tokyo, Japan). A custom anaerobic cuvette (1-cm light path, 5-ml sample volume) equipped with a combined platinum and Ag/AgCl electrode (6860-10C, Horiba, Tokyo, Japan) and a screw-capped side arm was used. Purified HLMW*b*_5 _sample or its site-specific mutants (final, 15 μM) either in the presence or absence of poly-L-lysine (200 μM) in 50 mM sodium-phosphate buffer (pH 7.0) was mixed with redox mediators (anthraquinone-2,6-disulfonate, 20 μM; 1,2-naphthoquinone, 20 μM; phenazine methosulfate, 20 μM; duroquinone, 20 μM; 2-hydroxy-1,4-naphtoquinone, 20 μM; riboflavin, 20 μM). For the redox measurements of the full-length form of human cytochrome *b*_5_, 50 mM sodium-phosphate buffer (pH 7.0) containing 0.5% (v/v) Triton X-100 was used as the buffer. The sample was kept under a flow of moistened Ar gas to exclude dioxygen and was continuously stirred with a small magnetic stirrer (CC-301, SCINICS, Tokyo, Japan) inside. Reductive titration was performed at 25°C by addition of small aliquots of sodium dithionite (4 or 16 mM) solution through a needle in the rubber septum on the side arm; for a subsequent oxidative titration, potassium ferricyanide (4 or 16 mM) was used as the titrant. In an appropriate interval, visible absorption spectra and redox potentials were recorded. The changes in absorbance (A555.0 *minus *A565.6; the peak in reduced form *minus *isosbestic point of HLMW*b*_5_) were corrected considering the dilution effect and analyzed with Igor Pro (v. 6.03A2) employing a Nernst equation with a single redox component.

## Results

### Purification of soluble domain of human cytochrome *b*_5 _(HLMW*b*_5_) and its mutants

Purification of HLMW*b*_5 _and its site-specific mutants was successful except for L51T mutant. Failure of purification for the L51T mutant was due to the inability to obtain a heme-bound holo-form. We confirmed that enough amounts of the protein corresponding to HLMW*b*_5 _was produced in *E. coli *cells upon addition of IPTG based on the SDS-PAGE analysis and CBB-250 staining. Addition of excess amounts of heme solution during the disruption of the *E. coli *cells to reconstitute the holo-form was unsuccessful, suggesting that the heme-pocket of the L51T mutant was perturbed significantly and not suitable for the accommodation of the heme prosthetic group, leading to the denatured form. Thus, we did not pursue the L51T mutant further in the present study.

### Properties of soluble domain of human cytochrome *b*_5 _(HLMW*b*_5_) and its mutants

The purified HLMW*b*_5 _showed characteristic visible absorption spectra as a native form of cytochrome *b*_5 _by showing absorption peaks at 413 nm for oxidized form and at 555, 526, and 423 nm for reduced form (spectra not shown). Purified HLMW*b*_5 _showed a single protein-staining band (CBB-250 staining) upon SDS-PAGE (12% gel) analysis with an apparent molecular size of 16.5 kDa. This value was, however, much larger than the expected value (13548.91 Da) for the NH_2_-terminal extension (20 amino acid residues, containing the 6x-His-tag moiety) *plus *the soluble domain (1-99 aa) of human cytochrome *b*_5_. To clarify the biochemical nature of the HLMW*b*_5_, we conducted MALDI-TOF-MS analyses. Untreated HLMW*b*_5 _sample showed a single peak at 13418 m/z corresponding to a mono-protonated form. A doubly-protonated form showed a weak peak at 6709 m/z. This result suggested that a post-translational modification (*i.e.*, removal of the initial Met residue) had occurred in HLMW*b*_5_. MALDI-TOF-MS analyses on the tryptic peptides of HLMW*b*_5 _(data not shown) proved that the Met residue at the initiation site was missing. We concluded that the purified HLMW*b*_5 _protein is a form with the sequence corresponding to 2-119 aa of HLMW*b*_5 _(theoretical molecular weight; 13471.72 Da).

All the purified mutants showed very similar UV-visible absorption spectra with those of HLMW*b*_5_, indicating that those site-specific mutations around the heme-binding pocket (except for the L51T mutant) did not affect significantly on the coordination or the electronic structure of the heme moiety.

### EPR spectroscopy of HLMW*b*_5 _and its mutants

**Figure 2:**
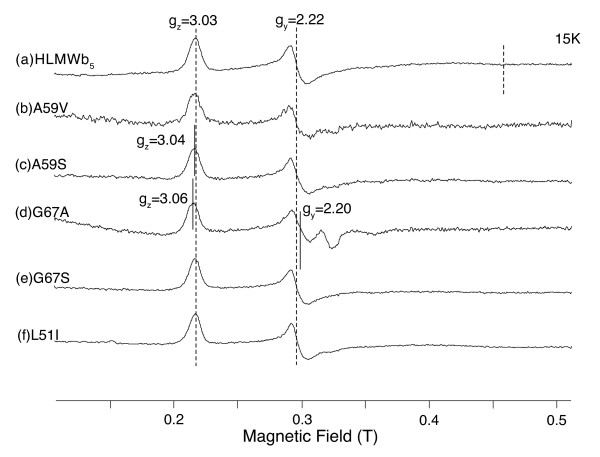
**X-band EPR spectrum of oxidized HLMW*b*_5 _measured at 15K and effects of the mutations on the spectrum**. Following samples in oxidized form in 50 mM sodium phosphate buffer pH 7.0 were frozen at 77K and their respective EPR spectrum was measured at 15K. HLMW*b*_5 _(trace **a**, 0.50 mM); A59V (trace **b**, 0.12 mM); A59 S (trace **c**, 0.19 mM); G67A (trace **d**, 0.20 mM); G67 S (trace **e**, 0.24 mM), and L51I (trace **f**, 0.27 mM). Ordinate of each spectrum was normalized appropriately based on the concentration for an easier visualization. Other conditions are described in the text. The signal around g = 2 in G67A mutant (**d**) was due to a contaminant from EPR tube.

The EPR spectrum of oxidized HLMW*b*_5 _measured at 15K showed g_z _= 3.03, g_y _= 2.22, and g_x _= 1.43 (Figure 2A; trace **a**), very close to those reported for rat [38], rat outer mitochondrial membrane (OM) [39] and pig [40] cytochromes *b*_5 _and human LMW*b*_5 _[32] in which the 6xHis-tag sequence (20 aa) at the NH_2_-terminal region is not present, or human erythrocyte cytochrome *b*_5 _[41]. However, it was slightly different from the report for the recombinant human erythrocyte cytochrome *b*_5 _(g_z _= 3.06, g_y _= 2.22, and g_x_= 1.42) [42]. It must be noted that there was no high-spin signals around g~6 nor the signals from adventitiously bound non-heme iron at g = 4.3 in the spectra (spectra not shown) [38].

All the purified mutants showed very similar EPR spectra to that of HLMW*b*_5 _as shown in Figure 2A. Closer examinations indicated that G67A mutant showed a slight perturbation on its heme coordination by showing g_z _= 3.06 and g_y _= 2.20, close to the values for house fly cytochrome *b*_5 _[43]. These results confirmed that the site-specific mutations introduced around the heme-binding pocket to modulate the hydrophobicity did not affect significantly on the coordination or the electronic structure of the heme prosthetic group.

For HLMW*b*_5 _and the G67A mutant, effects of the addition of poly-L-lysine (final concentration, 400 μM) on the EPR spectrum were examined. However, there was no apparent shift of their respective g-values (spectra not shown).

### Cyclic voltammetry of LMW***b***_5 _and its mutants

**Figure 3:**
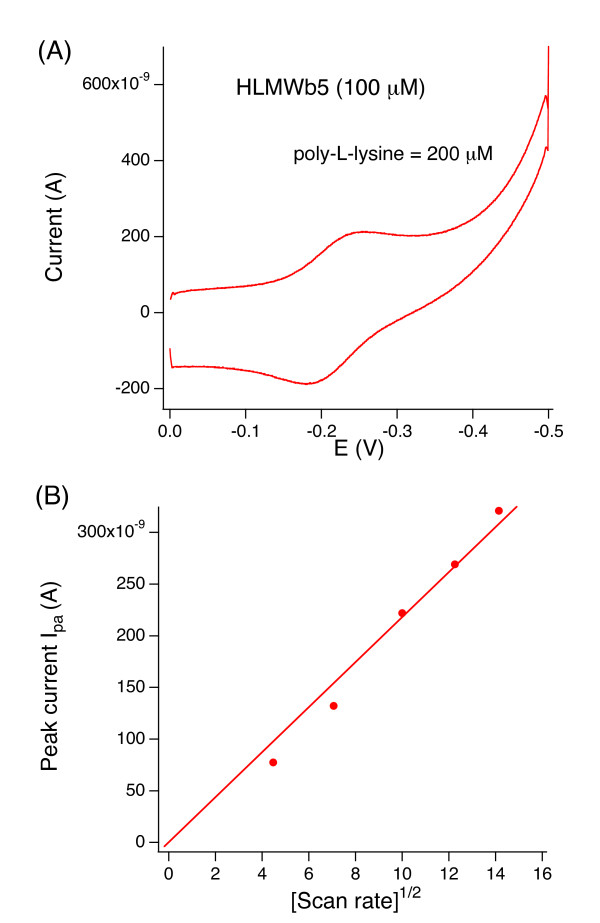
**Cyclic voltammogram of HLMW*b*_5 _in 50 mM sodium phosphate buffer pH 7.0**. (Panel **A**) The gold electrode was modified with β-mercaptopropionic acid and the voltammogram of HLMW*b*_5 _(100 μM (final) in 50 mM sodium phosphate buffer pH 7.0) was obtained in the presence of 200 μM of poly-L-lysine. The potential shown is *vs*. an Ag/AgCl reference electrode with an internal filling solution of 3 M KCl saturated with AgCl (*E*° = +197 mV *vs*. SHE). Scan rate = 100 mV/sec. (Panel **B**) Plot of the anodic peak current I_pa _against the square root of the scan rate ν_1/2_.

The Au electrode pre-treated with 3-mercaptopropionic acid gave reversible voltammetric responses for the HLMW*b*_5 _solution but only in the presence of poly-L-lysine. Without poly-L-lysine, there was no peak current. At least 50 μM of poly-L-lysine was required to observe a stable peak current (data not shown). In Figure 3A, a typical voltammogram for HLMW*b*_5 _in the presence of 200 μM of poly-L-lysine is shown. A plot of the square root of the scan rate *vs*. peak current (I_pa_) (or I_pc_, result not shown) was linear for scan rates up to and greater than 200 mV/sec (Figure 3B), indicating a diffusion-controlled reaction. The half-wave potential (corresponding to the midpoint potential) was estimated as -19.5 mV (*vs*. SHE), which was close to the values for the full-length human cytochrome *b*_5 _(-20.5 mV) and LMW*b*_5 _without the 6xHis-tag moiety (-21 mV) [32] and for bovine liver cytochrome *b*_5 _(-6 mV, -14 mV) [44] measured under similar experimental conditions (Table 1). These results indicated that presence of 6xHis-tag moiety or COOH-terminal hydrophobic transmembrane segment does not affect significantly on the redox properties of the hydrophilic heme-binding domain of HLMW*b*_5_. However, it must be noted that, in the case of full-length human cytochrome *b*_5 _(-20.5 mV), we observed relatively large peak separation values and, more significantly, the plot of the square root of the scan rate *vs*. peak current was not clearly linear. This might be due to the presence of detergent Triton X-100 (0.5~1.0%), which may interfere the smooth diffusion of cytochrome *b*_5 _molecules at the electrode surface by forming micelles with the COOH-terminal hydrophobic segments incorporated.

**Table 1:** Half-wave potentials of HLMW*b*_5_ and its site-specific mutants in comparison with various animal cytochrome *b*_5_ and their site-specific mutants.

Samples	half-wave potential (mV) (*vs*. SHE)	Electrode	references
HLMW*b*_5_	-19.5	Au*	present study
LMW*b*_5_	-21	Au*	[32]
full-length human cyt. *b*_5_	-20.5	Au*	present study
L51I	-30.5	Au*	present study
A59V	-29	Au*	present study
A59S	-31.5	Au*	present study
G67A	-40.5	Au*	present study
G67S	-32	Au*	present study
human erythrocyte cyt. *b*_5_	-9	Au**	[42]

			
rat OM cyt. *b*_5 _(soluble domain)	+8	Au*	[25]
rat OM cyt. *b*_5 _(soluble domain)	-40	Au*+Mg^2+^	[25]
rat OM cyt. *b*_5 _(soluble domain)	-78	Au*+Cr^3+^	[25]
rat OM cyt. *b*_5 _(soluble domain)	-27	Carbon	[28]
DiMe OM cyt. *b*_5 _(soluble domain)	+20	Carbon	[28]
V61L/V45L	-14	Carbon	[28]
rat OM cyt. *b*_5 _(soluble domain)	-26	ITO	[29]
DiMe OM cyt. *b*_5 _(soluble domain)	+4	ITO	[29]
V61I/V45I	-24	ITO	[29]
rat liver cyt. *b*_5 _(soluble domain)	+16.2	Au*^2^	[38]
A67V (soluble domain)	-2.8	Au*^2^	[38]
rat liver cyt. *b*_5 _(soluble domain)	-7	Au*^3^	[47]
bovine liver cyt. *b*_5 _(tryptic fragment)			
+ 20 mM Mg^2+^	-6	Au*^4^	[44]
bovine liver cyt. *b*_5 _(tryptic fragment)			
+ 20 mM Cr(NH_3_)_6_^3+^	-14	Au*^4^	[44]
bovine liver cyt. *b*_5 _(tryptic fragment)	-10	Au*^3^	[18]
V61E (bovine liver, tryptic)	-25	Au*^3^	[18]
V61Y (bovine liver, tryptic)	-33	Au*^3^	[18]
V61H (bovine liver, tryptic)	+11	Au*^3^	[18]
V61K (bovine liver, tryptic)	+17	Au*^3^	[18]
V45Y	-35	Au*^3^	[48]
V45H	+8	Au*^3^	[48]
V45E	-26	Au*^3^	[48]

The half-wave potentials (E_1/2_) were measured from respective cyclic voltammogram using various electrodes pre-treated as indicated.

Au*, gold-electrode modified with β-mercaptopropionic acid + poly-L-lysine (200 μM) carbon, DDAB-modified glassy carbon electrode

ITO, indium-doped tin oxide electrode + poly-L-lysine (200 μM)

Au**, gold-electrode modified with KCTCCA peptide

Au*^2^, gold-electrode modified with HO(CH_2_)_4_SH

Au*^3^, gold-electrode modified with cysteine

Au*^4^, gold-electrode modified with HSCH_2_COOH

**Figure 4:**
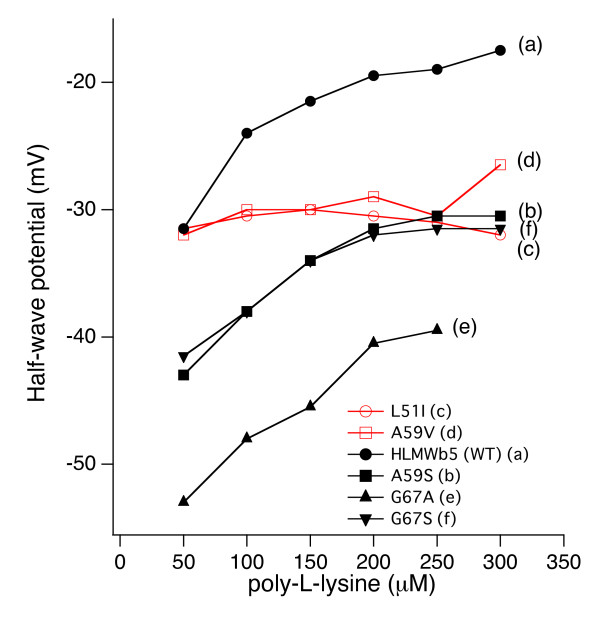
**Dependency of the half-wave potential (E_1/2_) of HLMW*b*_5_, A59 S, A59V, L51I, G67A, and G67 S mutants on the concentration of poly-L-lysine**. Titration was conducted using the gold electrode modified with β-mercaptopropionic acid and the scan rate was maintained at 100 mV/sec. The peak to peak separation of the cyclic volatmmograms throughout the titration was around 67 mV. Line (a), HLMW*b*_5 _(WT); line (b), A59 S, line (c), L51I; line (d), A59V; line (e), G67A; line (f), G67 S.

As noted previously, the voltammetric response of outer mitochondrial membrane (OM) cytochrome *b*_5 _measured by the Au electrode pre-treated with 3-mercaptopropionic acid (or similar thiol-containing reagents) were very dependent on the concentration of multivalent ions in the sample solution [25]. ‪It was postulated that multivalent cations could bind to the protein surface and to the electrode surface simultaneously and allow the negatively charged protein to approach the negatively charged electrode [25]. This phenomenon was termed as "ion gating" [45]. Therefore, we conducted detailed analyses concerning the dependency of half-wave potential (E_1/2_) of HLMW*b*_5 _on the concentration of poly-L-lysine in a range of 50~300 μM (Figure 4). Results showed that half-wave potential (E_1/2_) shifted in the positive direction as the concentration of poly-L-lysine increased and, around 200 μM of poly-L-lysine, it reached a plateau with a value about -20 mV (Figure 4 line (a)).

Rivera *et al*. reported that the electron transfer between the negatively charged electrode and the negatively charged OM cytochrome *b*_5 _was promoted by the addition of Mg^2+ ^or Ca^2+^, instead of poly-L-lysine [25]. However, in the present study, we could not observe any effects of Mg^2+ ^or Ca^2+ ^(~20 mM) to produce a reversible cyclic voltammogram of HLMW*b*_5_; rather it caused a precipitation of the protein in the sample solution. Therefore, we did not pursue further on the effects of these cations on the cyclic voltammogram in the present study.

We, then, measured the cyclic volatmmogram for the five site-specific mutants (L51I, A59V, A59 S, G67A, G67S) in the presence of poly-L-lysine in different concentrations (50~300 μM) and the apparent half-wave potentials (E_1/2_) were calculated (Figure 4; Table 1). A typical result for the A59 S mutant is shown in Figure 4 line (b). In this case, half-wave potential shifted positively as the concentration of poly-L-lysine increased and, at 200 μM of poly-L-lysine, it reached a plateau as observed for wild-type HLMW*b*_5 _(Figure 4 line (a)). The maximum value was around -30 mV. Similar concentration dependency was also observed for the G67 S and G67A mutants (Figure 4 lines (e) and (f)), although the G67A mutant showed a significant negative shift in its half-wave potentials (Figure 4 line (e)). It is noteworthy that the concentration required to reach a plateau was around 200 μM in most of the samples measured in the present study. This value was consistent with the previous proposal for the formation of the OM cytochrome *b*_5_-poly-L-lysine complex (1:2) [25]. However, for the L51I and A59V mutants, dependency of the half-wave potential on the poly-L-lysine concentration was not observed (Figure 4 lines (c) and (d)). In these two mutants, the half-wave potential was around -30 mV irrespective of the concentration of poly-L-lysine (Figure 4 lines (c) and (d)).

### Spectroscopic electrochemical titrations of HLMW*b*_5 _and its mutants

**Figure 5:**
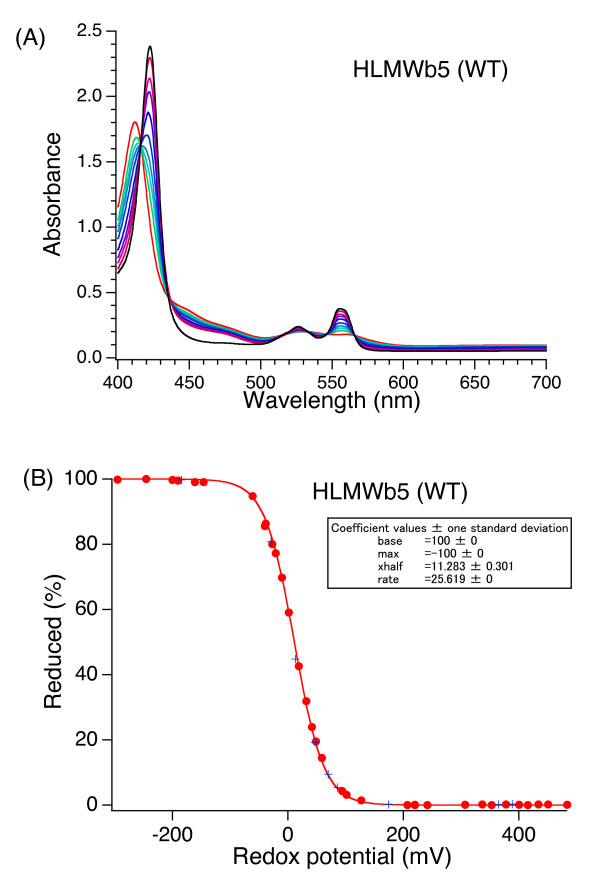
**Midpoint potential measurement of HLMW*b*_5 _with spectroelectrochemical titration**. Spectroelectrochemical titration was conducted by recording the absorption spectrum of HLMW*b*_5 _(15 μM in 50 mM sodium phosphate buffer pH 7.0) at various redox potentials by the addition of sodium dithionite to the oxidized form at 25°C in the presence of various redox mediators (for detail, see main text). Least-square curve-fitting of the spectroelectrochemical titration data by using the Nernst equation assuming a single redox component. Solid circles indicate data points for the reductive phase and + for the oxidative phase. Other conditions are indicated in the main text.

Spectroscopic redox behavior of HLMW*b*_5 _(Figure 5) showed a good agreement between the points obtained during reductive and oxidative titrations (Figure 5; solid circles for the reductive phase and × for the oxidative phase). The apparent midpoint potentials were estimated to be around 0 mV at pH = 7.0. Least square fitting analysis using the Nernst equation with a single redox component showed the midpoint potential as -3.2 mV (Figure 5; a solid curve fitted for solid circles), consistent with a previous report on human erythrocyte cytochrome *b*_5 _(-2 mV) determined by a similar method [46]. We also measured the midpoint potential for the full-length form of human cytochrome *b*_5 _(under an identical buffer condition but in the presence of 0.5% (v/v) Triton X-100) and found it as -2.6 mV (data not shown). This result confirmed that presence of 6xHis-tag sequence (20 aa) at the NH_2_-terminal region or COOH-terminal hydrophobic transmembrane segment does not affect significantly on the redox properties of the hydrophilic heme-binding domain of HLMW*b*_5_.

Midpoint potentials of the site-specific mutants were obtained similarly. The values were tabulated in Table 2. The lowest value was found for the L51I mutant; but all the midpoint potentials were found within a relatively narrow range of 7 mV difference. This fact indicated that the site-specific mutations introduced in the present study did not affect significantly on their static redox properties.

**Table 2:** Midpoint potentials of human and bovine cytochrome *b*_5_ and its site-specific mutants.

Samples	Midpoint potentials (mV) (*vs*. SHE)	method	References
HLMW*b*_5_	-3.2	optical titration	present study
HLMW*b*_5 _+ poly-L-lysine (200 μM)	+16.5	optical titration	present study
human erythrocyte cytochrome *b*_5_	-2	optical titration	[46]
full-length human cytochrome *b*_5_	-2.6	optical titration	present study
full-length human cytochrome *b*_5_			
+ poly-L-lysine (200 μM)	+8.7	optical titration	present study
L51I	-9.5	optical titration	present study
L51I + poly-L-lysine (200 μM)	+10.5	optical titration	present study
A59V	-7.7	optical titration	present study
A59V + poly-L-lysine (200 μM)	+11.7	optical titration	present study
A59S	-4.9	optical titration	present study
A59 S + poly-L-lysine (200 μM)	+9.6	optical titration	present study
G67A	-8.4	optical titration	present study
G67A + poly-L-lysine (200 μM)	-2.7	optical titration	present study
G67S	-7.3	optical titration	present study
G67S + poly-L-lysine (200 μM)	+14.2	optical titration	present study
			

bovine liver cyt. *b*_5 _(tryptic fragment)	+5.1	OTTLE	[49]
bovine liver cyt. *b*_5 _(tryptic fragment)	-1.8	OTTLE	[50]
bovine liver cyt. *b*_5 _(tryptic fragment)	+5	OTTLE	[44]
bovine liver cyt. *b*_5 _(tryptic fragment)			
+ 20 mM Mg^2+^	+15	OTTLE	[44]
bovine liver cyt. *b*_5 _(tryptic fragment)	+2	OTTLE	[24]
F35L	-26	OTTLE	[24]
F35H	-49	OTTLE	[24]
F35Y	-64	OTTLE	[24]
rat OM cyt. *b*_5 _(soluble domain)	-102	OTTLE	[25]
rat OM cyt. *b*_5 _(soluble domain)			
+ poly-L-lysine (104 μM)	-70	OTTLE	[28]
rat OM DiMe cyt. *b*_5 _(soluble domain)	-36	OTTLE	[28]
rat OM DiMe cyt. *b*_5 _(soluble domain)			
+ poly-L-lysine (104 μM)	-33	OTTLE	[28]
V61L	-117	OTTLE	[28]
V61L/V45L	-148	OTTLE	[28]
V61I/V45I	-63	OTTLE	[29]

Midpoint potentials of human cytochrome *b*_5 _(HLMW*b*_5_) and its mutants were estimated for the redox titration data obtained in the absence or presence of poly-L-lysine by a least-square curve fitting using the Nernst equation with assuming a single redox component. For a comparative purpose, midpoint potentials for the tryptic fragment of bovine liver cytochrome *b*_5 _and OM cytochrome *b*_5 _obtained by OTTLE method were presented.

OTTLE, optically-transparent-thin-layer-electrode in the presence of Ru(NH_3_)_6 _as a mediator

In the next stage, we examined the effect of addition of poly-L-lysine (final 200 μM) on the redox potentials of HLMW*b*_5 _and its site-specific mutants determined by a static equilibrium method. In the case of HLMW*b*_5_, the effect was evident (Figure 5B; solid squares for the reductive phase and + for the oxidative phase). The least square fitting analysis using the Nernst equation with a single redox component showed that the addition of poly-L-lysine caused a positive shift of its midpoint potential by ~20 mV (from -3.2 mV to +16.5 mV). Similar positive shifts of the midpoint potential upon addition of poly-L-lysine were found for all the samples examined in the present study including the full-length cytochrome *b*_5 _and five site-specific mutants (Table 2). It is noteworthy that the shifts were close to +20 mV except for the G67A mutant.

## Discussion

### Relative importance and roles of the three conserved residues

Three conserved hydrophobic amino acid residues (Leu51, Ala59, and Gly67) consisting of the heme-binding pocket of cytochrome *b*_5 _were not investigated in the past, despite of their relatively high conservation among the cytochrome *b*_5 _protein family (Figure 1A). The most significant effect of the mutation was observed for the L51T mutant, in which the heme-pocket moiety might be perturbed significantly and would not be suitable for the accommodation of a heme prosthetic group, leading to an apo-form (or a denatured form) when expressed in *E. coli *cells. Introduction of a hydrophilic Thr residue in the bottom of the hydrophobic heme-pocket might be too harsh to maintain the original native structure, suggesting the critical role of this hydrophobic residue (Figure 1B). Our computer modeling study indicated that the L51T mutant would have a larger cavity in the heme pocket above the heme plane, being consistent with this view (see Fig. S1(A and B); additional file 1). On the other hand, introduction of a Ser (or Ala) residue by replacing Gly67 residue did not cause such an effect within the heme-pocket, indicating that a hydrophilic residue at the entrance of the pocket might be tolerable and, therefore, did not cause significant influences (Figure 1B). Results of the computer modeling study were consistent with this view (see Fig. S1(A and C); additional file 1). Ala59 residue resides in the lowest bottom of the heme pocket. The computer modeling study indicated that substitution with Ser (or Val) did not cause any substantial change in the heme pocket as well. EPR spectra of the oxidized forms of these mutants (except for the L51T) showed, indeed, similar spectra with that of HLMW*b*_5 _(Figure 2). However, only for the G67A mutant, its EPR spectrum indicated a slight but distinct perturbation (g_z _= 3.06, g_y _= 2.20) (Figure 2), suggesting some important role(s) of Gly67 residue as an adjacent one to the axial His68 residue. As a whole, these observations indicated that the three conserved hydrophobic amino acid residues (Leu51, Ala59, and Gly67) were not particularly important in having direct interactions with the heme prosthetic group but were very important for maintaining the hydrophobic and structurally-organized environments around the heme prosthetic group. It might be noteworthy that naturally occurring human cytochrome *b*_5 _T60A mutant [12] displayed an enhanced susceptibility to proteolytic degradation, indicating the destabilized structure around its heme pocket.

### Cyclic voltammetry of cytochrome *b*_5_

In our present study, we observed a just reverse phenomenon reported for OM cytochrome *b*_5 _[25], in which the half-wave potential was about 110 mV higher than the midpoint potential determined by the equilibrating method (Table 1 &2). In our present case, the half-wave potential of HLMW*b*_5 _(-19.5 mV; in the presence of 200 μM of poly-L-lysine) was about 16 mV lower than the midpoint potential measured by an equilibrium method (-3.2 mV) (Table 1 &2), although the half-wave potential itself showed a positive shift as the concentration of poly-L-lysine was increased, as found for OM cytochrome *b*_5 _[25], reaching the plateau of -17.5 mV. A similar redox behavior to our HLMW*b*_5 _was reported previously for bovine liver cytochrome *b*_5 _tryptic fragment, in which midpoint potential determined by the equilibrating method (in the presence of 20 mM Mg^2+^) showed +15 mV, whereas the half-wave potential under a similar condition was -6 mV, leading to a negative shift of -21 mV (Table 1 &2) [44].

The difference between the half-wave potential and midpoint potential determined by the equilibrating method observed for bovine liver cytochrome *b*_5 _tryptic fragment was ascribed to the different surface properties of the electrodes used [44]. Following the proposal by Wang *et al*. [44], our present results can be explained reasonably. In the cyclic voltammetry, poly-L-lysine binds simultaneously with the protein moiety and the carboxy group of β-mercaptopropionic acid on the surface of the electrode. In the spectroscopic equilibrating method, poly-L-lysine binds only to the protein and the electron transfer occurs directly between the electrode and the protein. Therefore, in the cyclic voltammetry, the interaction of poly-L-lysine with the carboxylates of the electrode-coated β-mercaptopropionic acid decreased its effective density of positive charge and, therefore, the half-wave potential is more negative than those measured by the spectroscopic equilibrating method. Additionally, dehydration of the heme edge by excluding water from the complex interface might also contribute significantly on the positive shift of the half-wave potential [29].

However, the differences between the half-wave potential and midpoint potential determined by the equilibrating method were so much different each other among OM cytochrome *b*_5_, human cytochrome *b*_5_, and bovine liver cytochrome *b*_5_. This fact suggested that the exact mechanism for determining the redox potential is very complex. Reality might exist between the two simplified possibilities. The gross tertiary structures around the heme moiety would be conserved well among OM cytochrome *b*_5_, human cytochrome *b*_5_, and bovine liver cytochrome *b*_5 _(Figure 1B and 1C) and, therefore, the distributions of acidic residues on the surface of the heme domain are also well conserved (Figure 1A and 1C). Therefore, the proposed scheme for the formation of the complex between OM cytochrome *b*_5 _and poly-L-lysine occurs on the protein surface of HLMW*b*_5 _delineated by the exposed heme propionate and corresponding acidic residues (Glu49, Glu53, Glu61, and Asp65) as well. Therefore, slight conformational differences around the heme propionate group would be a very important factor for controlling the heme redox potentials.

### Effects of site-specific mutations within the heme pocket on the cyclic voltammetry

Other factor(s) important for the regulation of heme redox potential is the hydrophobicity around the heme pocket [29]. To evaluate such a hydrophobic effect within the heme pocket on the redox potential, we produced five site-specific mutants in expecting to have different modulations on the hydrophobicity. However, the midpoint potentials for these mutants showed only slight variations ranging from -5 to -9 mV. This result might be consistent with the results of our computer modeling study, which indicated that the site-specific mutants did not cause any substantial changes in the heme pocket except for the L51T mutant (see Fig. S1(A and B); additional file 1).

On the other hand, the half-wave potentials for these mutants showed a much larger variation (-29~-43 mV) and a more negative value than that of HLMW*b*_5 _(-19.5 mV). More interestingly, the half-wave potentials for these mutants were categorized into two groups, one showing clear dependency on the poly-L-lysine concentration (HLMW*b*_5_, A59 S, G67A, and G67S), and the other showing independency on the poly-L-lysine concentration (L51I and A59V) (Figure 4). The curvature of the titration curves for those showing the dependency on the poly-L-lysine concentration was somewhat similar each other (Figure 4), indicating a similar mechanism for controlling the redox potential being operative within those. Therefore, for these mutants, very similar interactions between poly-L-lysine and the protein surface of HLMW*b*_5 _delineated by the exposed heme propionate and the acidic residues (Glu49, Glu53, Glu61, and Asp65) (Figure 1C) might occur, as proposed originally for rat OM cytochrome *b*_5_. Following this scenario, one may argue that the large variation in the half-wave potential might be ascribed to the difference in the dehydration around the heme moiety upon the complex formation with poly-L-lysine [29]. On the other hand, the mutants showing an independency on the poly-L-lysine concentration (*i.e*., L51I and A59V) might be reflecting the difference in microenvironment around the heme propionate group itself caused by the slight change in the heme cavity structure. Alternatively, since both Leu51 and Ala59 locate in the bottom of the heme cavity (Figure 1B), slight conformational change upon the mutations might propagate to the local negative surface structure around Glu49, Glu53, Glu61, and Asp65 (Figure 1C), resulting in the independency on the poly-L-lysine concentration. However, our computer modeling study did not support any of these possibilities, indicating the limitation of this kind of modeling study.

One may argue about the cause of the significant negative shift in the half-wave potential of the G67A mutant (Figure 4 line (e); Table 1). The likely explanation for the negative shift would be a change in the hydrophobicity within the heme-pocket. But we should not exclude the possibility of a slight structural change caused by the replacement. Indeed, the G67A mutant showed a distinct negative value compared to HLMW*b*_5 _in the midpoint potential measurement as well (Table 2). However, the G67 S mutant, that might be expected to cause just a reverse of the G67A mutant, actually showed an intermediate value between those of HLMW*b*_5 _and the G67A mutant. Therefore, the significant negative shift would be caused not only by changes in the hydrophobicity but by other factors including changes in the heme coordination (as evidenced by the slight shifts of g-values in its EPR spectrum) (Figure 2 trace **d**). Further, the binding mode of poly-L-lysine itself might be altered due to a slight change in local negative surface structure, resulting in lowering of the dehydration effect upon the complex formation at the heme edge [29].

### Correlations between the half-wave potential and midpoint potential

**Figure 6:**
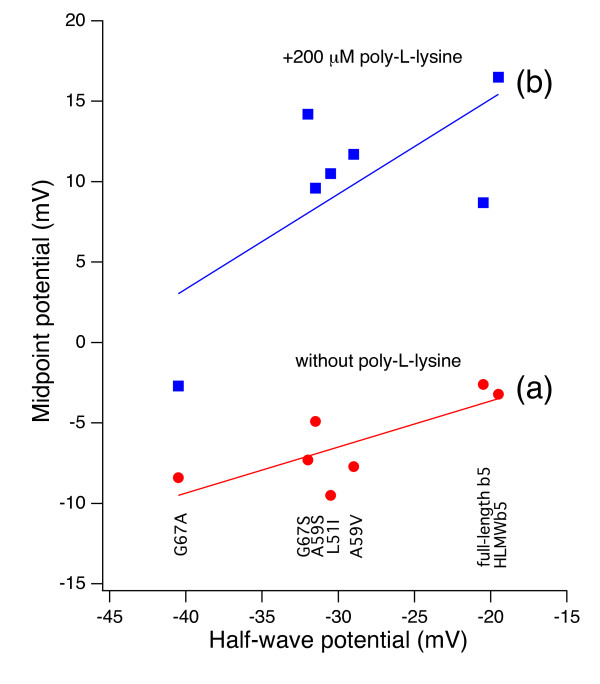
**Correlations between the midpoint potential and half-wave potential (E_1/2_) for HLMW*b*_5 _and its site-specific mutants**. The half-wave potentials (E_1/2_) were measured at a gold electrode modified with β-mercaptopropionic acid in the presence of 200 μM of poly-L-lysine at 25°C as described in the main text. Midpoint potentials were measured in the absence (line (**a)**) or presence (line (**b)**) of 200 μM of poly-L-lysine as described in the main text. Lines were drawn to show the correlations assuming a linear function (f(x) = bx + a). Calculated coefficients and standard deviations are a = 2.945 ± 3.13, b = 0.2863 ± 0.105 for line (**a**) (with Pearson product-moment correlation coefficient = 0.768 and paired student's t-test value, **P < 0.01), and a = 26.946 ± 8.17, b = 0.59028 ± 0.274 for line (**b**) (with Pearson product-moment correlation coefficient = 0.706 and paired student's t-test value, **P < 0.01).

Interestingly, when the midpoint potential measured in the absence of poly-L-lysine was plotted against the half-wave potential for each of HLMW*b*_5 _and mutants, there was a good correlation between these two values (Figure 6 line **a**), in which the former were always 16~32 mV more positive than the latter. When the midpoint potential measured in the presence of poly-L-lysine (200 μM) was plotted against the half-wave potential similarly, there was a good correlation as well, in which the midpoint potential values were further up-shifted by 10~20 mV (Figure 6 line **b**). This fact suggested that both the binding of poly-L-lysine and the changes of the hydrophobicity around the heme moiety (both within the heme-pocket and the exposed heme edge) regulate the half-wave potential of cytochrome *b*_5 _and that the overall redox potentials were modulated by both factors in similar extents.

## Conclusions

Present study showed that simultaneous measurements of the midpoint potential and the half-wave potential could be a good evaluating methodology for the analyses of static and dynamic redox properties of various hemoproteins, including cytochrome *b*_5_, if we took them with an appropriate precaution. In the actual biological electron transfer, the reduction potential of cytochrome *b*_5 _might be modulated differently upon the formation of a transient complex with a partner protein (cytochrome *c*, hemoglobin, or cytochrome *b*_5 _reductase). The modulations might be mediated by a gross conformational change in the tertiary structure, by a slight change(s) in the local structure including surface charges, or by the change(s) in the hydrophobicity around the heme moiety (both within the heme-pocket and the exposed heme edge), as found for the interaction with poly-L-lysine. Therefore, the system consisting of cytochrome *b*_5 _and its partner protein(s) or small peptide(s) might be a good paradigm for the study of biological electron transfer reactions.

## List of abbreviations used

Abbreviations used are: LMW*b*_5_: human liver microsomal cytochrome *b*_5 _soluble domain (amino acid residues from Met1 to Leu99); HLMW*b*_5_: human liver microsomal cytochrome *b*_5 _soluble domain with an additional extension of the sequence of MGSSHHHHHHSSGLVPRGSH at the NH_2_-terminus of the LMW*b*_5 _protein; EPR: electron paramagnetic resonance; OM: outer mitochondrial membrane; MALDI-TOF: matrix-assisted laser desorption ionization-time of flight; SHE: standard hydrogen electrode.

## Competing interests

The authors declare that they have no competing interests.

## Authors' contributions

This study was designed and supervised by FT and MT. Experiments were performed by AT and YS. Analysis of the data was performed by AT, YS, MM and MT. EPR experiments and the data analysis were performed by HH. MT drafted the manuscript and all authors read and approved the final version.

## Supplementary Material

Additional file 1 **Results of Computer Modeling Study**. A computer modeling study for the three-dimensional structure of human cytochrome *b*_5 _using the coordinate of an NMR solution structure (PDB code: 2I96; model 1) Click here for file
