# Supplementary material for: Direct electrochemical analyses of human cytochromes b5 with a mutated heme pocket showed a good correlation between their midpoint and half wave potentials
Source: J Biomed Sci. 2010 Dec 4;17(1):90. doi: 10.1186/1423-0127-17-90 (PMC3014896; doi:10.1186/1423-0127-17-90)

Additional File 1

Results of Computer Modeling Study

Methods:

We conducted a computer modeling study for the three-dimensional structure of human cytochrome b_5 using the coordinate of an NMR solution structure (PDB code: 2I96; model 1) by employing softwares "Coot" (GNU GPL), "CCP4MG" (v.2.4.2) (University of York, England), and "MolFeat" (v.4.5)(FiatLux Corp., Tokyo, Japan). For evaluating the effects of the mutations, the three conserved hydrophobic residues around the heme pocked were each replaced with Ile, Thr (for Leu51), Val, Ser (for Ala59), Ala, and Ser (for Gly67) by employing "Coot". Then, the orientation of the side-chain group of the introduced residue was re-adjusted to make an appropriate hydrogen bond net-work with surrounding residues, but, in the absence of the heme prosthetic group. The molecular surface was, then, calculated based on the Van der Waals radius, and was drawn with a usual coloring scheme for the electrostatic potential (*i.e.*, blue, basic; white, neutral; red, acidic). Finally, the heme group with a ball-stick model was fitted into the calculated model. In all the figures, the view direction was fixed in the same orientation with that of Figure 1(A) of the main text for the visual purpose.

Results:

In the present study, we also performed a computer modeling study for the 3D structure of human cytochrome b_5 using the coordinate of an NMR solution structure. Since we did not perform any MD calculations for the mutated structure, changes in a total volume of the heme pocket upon the mutations were largely dependent on the difference in the volume of the two concerning side-chain groups. However, if we focused on a possible local structural change around the heme pocket, there might be some meaningful observations. Fig. S1(A) and (B) showed such typical examples. In Fig. S1(B), the calculated surface of the heme pocket for the L51T mutant indicated a much larger cavity in the bottom of the hydrophobic pocket compared to that of wild-type structure (A). This was primarily due to the local structural change caused by

the reorganization of a hydrogen-bond net-work within the heme pocket, rather than the slight lowering of the side-chain volume upon the mutation. Such enlargement of the heme cavity in the bottom of the pocket would allow an entry of water molecules or cause the perturbation of the contacts between the heme prosthetic group and the surrounding hydrophobic residues, leading to the destabilization of the bound heme prosthetic group. This might be the reason for the failure to obtain the holo-form for the L51T mutant in the present study, as discussed in the main text. On the other hand, the A59S mutant did not show such a structural change in the heme pocket due to the absence of significant re-organization of the hydrogen-bond net-work upon the mutation (not shown in the figure). In the case of the G67S mutant, the changes occurred around the entrance of the heme pocket (Fig. S1(C)). Therefore, there was no structural change within the major part of the heme pocket itself. For other three conservative mutations (L51I, A59V, and G67A), there were no appreciable structural changes both in the hydrophobic heme cavity and in the molecular surface (not shown in the figure).

Figure S1. Evaluation of the mutations around the heme pocket of human cytochrome b_5 by computer modeling. (A) Wild-type, (B) L51T, (C) A59S. Asterisks (*) in panels (B) and (C) indicate the mutation sites.

(A)

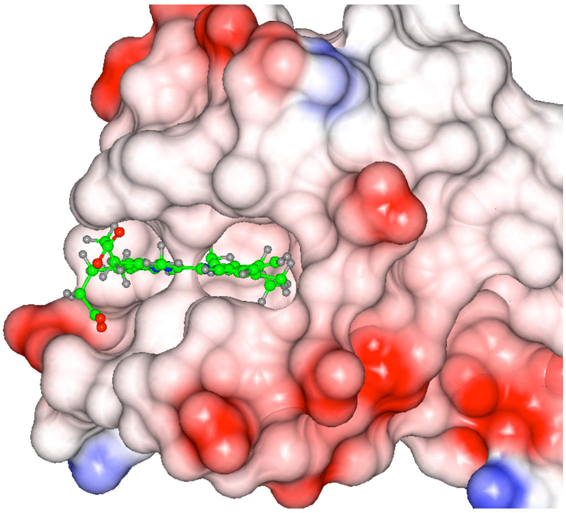

(B)

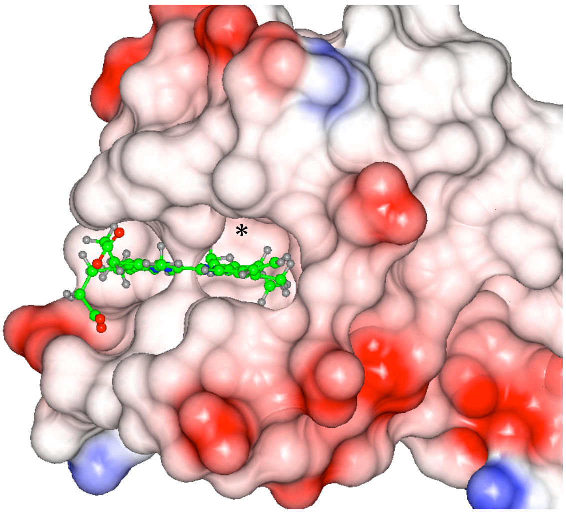

(C)

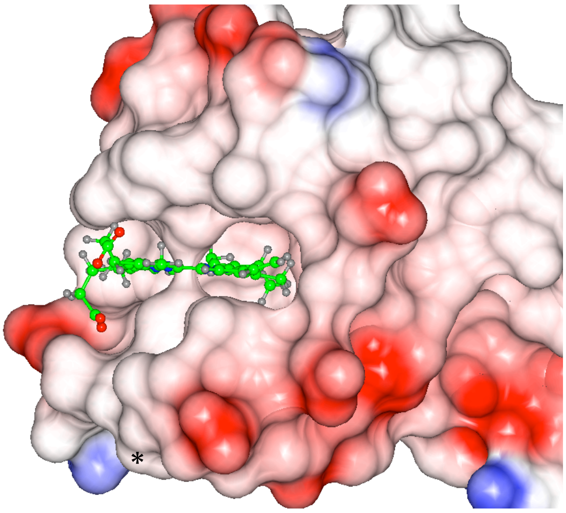

Supplement: Additional file 1 — Results of Computer Modeling Study. A computer modeling study for the three-dimensional structure of human cytochrome b5 using the coordinate of an NMR solution structure (PDB code: 2I96; model 1) [file 1423-0127-17-90-S1.PDF]
